# The Role of NLRP3 Inflammasome Activities in Bone Diseases and Vascular Calcification

**DOI:** 10.1007/s10753-020-01357-z

**Published:** 2020-11-20

**Authors:** Chenyang Yu, Caihua Zhang, Zhihui Kuang, Qiang Zheng

**Affiliations:** 1 Department of Orthopedic Surgery, The Second Affiliated Hospital, School of Medicine, Zhejiang University, No. 88, Jiefang Road, Hangzhou, 310009 China; 2 Orthopedics Research Institute of Zhejiang University, No. 88, Jiefang Road, Hangzhou, 310009 China

**Keywords:** inflammation, NLRP3 inflammasome, bone remodeling, osteolysis, vascular calcification

## Abstract

Continuous stimulation of inflammation is harmful to tissues of an organism. Inflammatory mediators not only have an effect on metabolic and inflammatory bone diseases but also have an adverse effect on certain genetic and periodontal diseases associated with bone destruction. Inflammatory factors promote vascular calcification in various diseases. Vascular calcification is a pathological process similar to bone development, and vascular diseases play an important role in the loss of bone homeostasis. The NLRP3 inflammasome is an essential component of the natural immune system. It can recognize pathogen-related molecular patterns or host-derived dangerous signaling molecules, recruit, and activate the pro-inflammatory protease caspase-1. Activated caspase-1 cleaves the precursors of IL-1β and IL-18 to produce corresponding mature cytokines or recognizes and cleaves GSDMD to mediate cell pyroptosis. In this review, we discuss the role of NLRP3 inflammasome in bone diseases and vascular calcification caused by sterile or non-sterile inflammation and explore potential treatments to prevent bone loss.

## INTRODUCTION

**Fig. 1:**
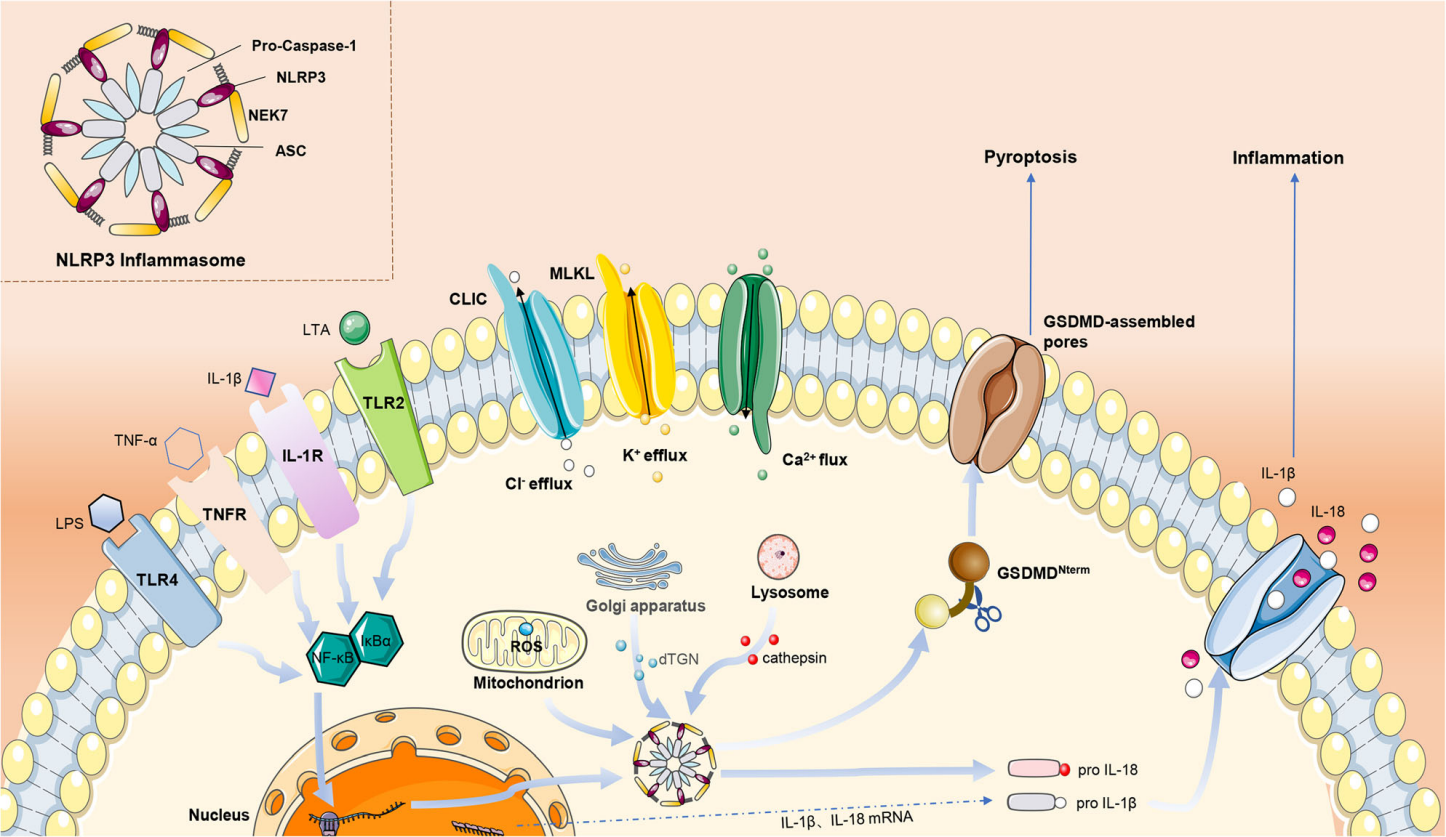
Mechanisms of activation of the NLRP3 inflammasome. A canonical NLRP3 gene transcription is initiated by PRR including TLRs, TNFR, and IL-1R. PRR is activated by the corresponding PAMPs and phosphorylated IκB, followed by release of NF-κB into the nucleus to initiate expression of NLRP3 gene. The outflow of K^+^, Cl^−^, and influx of Ca^2+^ activates the NLRP3 inflammasome. Mitochondria continually produce ROS and are the major source of cellular ROS. The phagocytosis of the lysosome on the particles will lead the lysosome to rupture and release the cathepsin into the cytoplasm. NLRP3 activators were found to promote trans-Golgi network disassembly into dTGN. The PtdIns4P on dTGN recruits NLRP3 and promotes NLRP3 aggregation. Activated NLRP3 inflammasome cleaves pro-caspase-1 into caspase-1. Caspase-1 promotes the maturation and release of pro-IL-1β and pro-IL-18 to induce inflammation and cleaves GSDMD to form GSDMD-assembled pores in the cell membrane, thereby triggering cell pyroptosis.

Inflammasomes play a vital role in inflammatory responses. They activate immune responses by releasing cytokines and inducing pyroptosis [1, 2]. The NOD, LRR, and pyrin domain-containing protein 3 (NLRP3) is an essential inflammasome of immune response and can be activated by various stimuli [3]. The inflammasome contains NLRP3, apoptosis-associated speck-like protein containing a CARD (ASC) and pro-caspase-1 [4]. Activated NLRP3 can cleave pro-caspase-1 into p20 and p10 subunits, inducing the maturation and release of pro-inflammatory cytokines such as interleukin-1β (pro-IL-1β) and IL-18 [5]. IL-1β can induce secretion of RANKL (receptor activator of NF-κB ligand) and activate osteoclasts, causing a series of inflammatory responses [6]. Activated caspase-1 specifically recognizes and cleaves gasdermin D (GSDMD) causing cell pyroptosis [7, 8]. It is currently believed that both pathogen-associated molecular patterns (PAMPs) and damage-associated molecular patterns (DAMPs) can activate the innate immune system and inflammatory responses through the pattern recognition receptor (PRR). Common PAMPs include lipopolysaccharide (LPS), lipoteichoic acid (LTA), peptidoglycan (PGN) [9], viral double-stranded RNA (dsRNA) [10], and others. DAMPs include misfolded or aggregated proteins, metabolites and wear fragments of prosthetic implants. Furthermore, several cytokines such as IL-1β, interferon (IFN), and tumor necrosis factor (TNF) are described as “inducible DAMPs and conditional DAMPs” [11, 12]. The PAMPs and DAMPs can be recognized by the corresponding PRR, which activates downstream signaling pathways causing inflammation or antibacterial responses. The expression of NLRP3 inflammasome requires at least two steps. (1) Priming the transcription of the NLRP3 gene. Initiation is achieved through activation of PRR of nuclear factor kappa-B (NF-κB) including toll-like receptors (TLRs), interleukin-1 receptor (IL-1R), and tumor necrosis factor receptor (TNFR) [13, 14]. The transcriptionally active form of NF-κB is predominantly a heterodimer formed through the combination of p65 and p50. NF-κB and IκB (inhibitor of NF-κB) form a trimer in the cytoplasm. When IκB phosphorylation is activated, NF-κB is released into the nucleus [15]. As a result, the expression of the inflammasome components NLRP3, caspase-1, and pro-IL-1β is upregulated. Before activation, NLRP3 needs to be bound to NEK7 by bridging adjacent NLRP3 subunits with bipartite interactions [16]. (2) Activation of NLRP3. The specific molecular mechanism of activation is still unclear, but there are several NLRP3 inflammasome activation models: (1) certain NLRP3 activators such as MLKL and P2X7 form pores in the plasma membrane, causing K+ efflux [17]; (2) chloride intracellular channel (CLIC) can also activate NLRP3 by promoting Cl− outflow [18, 19]; (3) in addition to lysosomal rupture [20] and mitochondrial dysfunction [21], metabolic dysfunction can activate NLRP3 inflammasome [22]; (4) trans-Golgi was found to be disassembled to form vesicles called dispersed trans-Golgi network (dTGN). The phospholipid phosphatidylinositol-4-phosphate (PtdIns4P) on dTGN can combine with NLRP3 and promote the latter to aggregate, thereby making NLRP3 activated [23]. Inflammasomes play a crucial role in numerous cell types including myeloid cells, osteoclasts, osteoblasts, and vascular endothelial cells. However, the role of NLRP3 inflammasome in bone disease and vascular calcification is still unclear (Fig. 1).

Osteopenia is a disease characterized by decreasing bone mass, increasing bone fragility, and a tendency of fracture. The internal mechanism involves imbalance between bone formation and bone resorption. Excessive bone resorption results in low bone density. The key factors that contribute to bone loss are reduced osteogenic differentiation of stem cells and mature osteoclasts. The key transcription factors of osteogenesis such as runt-related transcription factor 2 (RUNX2), Osterix, and osteogenically related proteins including osteopontin (OPN), osteocalcin (OCN), and bone morphogenetic protein (BMP2) are upregulated, indicating that osteogenic differentiation has occurred in bone marrow mesenchymal stem cells (BMSCs). The formation of osteoclasts requires macrophage colony-stimulating factor (M-CSF) and receptor activator of RANKL. Preosteoclasts are fully differentiated by recruitment of TNF receptor–related factor 6 (TRAF6) after the stimulation of RANKL [24].

Bone loss is manifested in not only osteoporosis but also a change in bone homeostasis which can occur in osteomyelitis, arthritis, and periodontitis. Numerous autoinflammatory diseases can cause bone destruction or arthritis because the process of bone remodeling is regulated by inflammatory response. In addition, joint replacement surgery is increasingly being applied clinically to treat fractures or arthritis. However, a few patients experience aseptic loosening. Biological factors primarily include sterile inflammatory response caused by the prosthetic material and its wear particles. The NLRP3 inflammasome regulates bone remodeling and inflammation around the prosthesis through inflammatory factors.

**Fig. 2:**
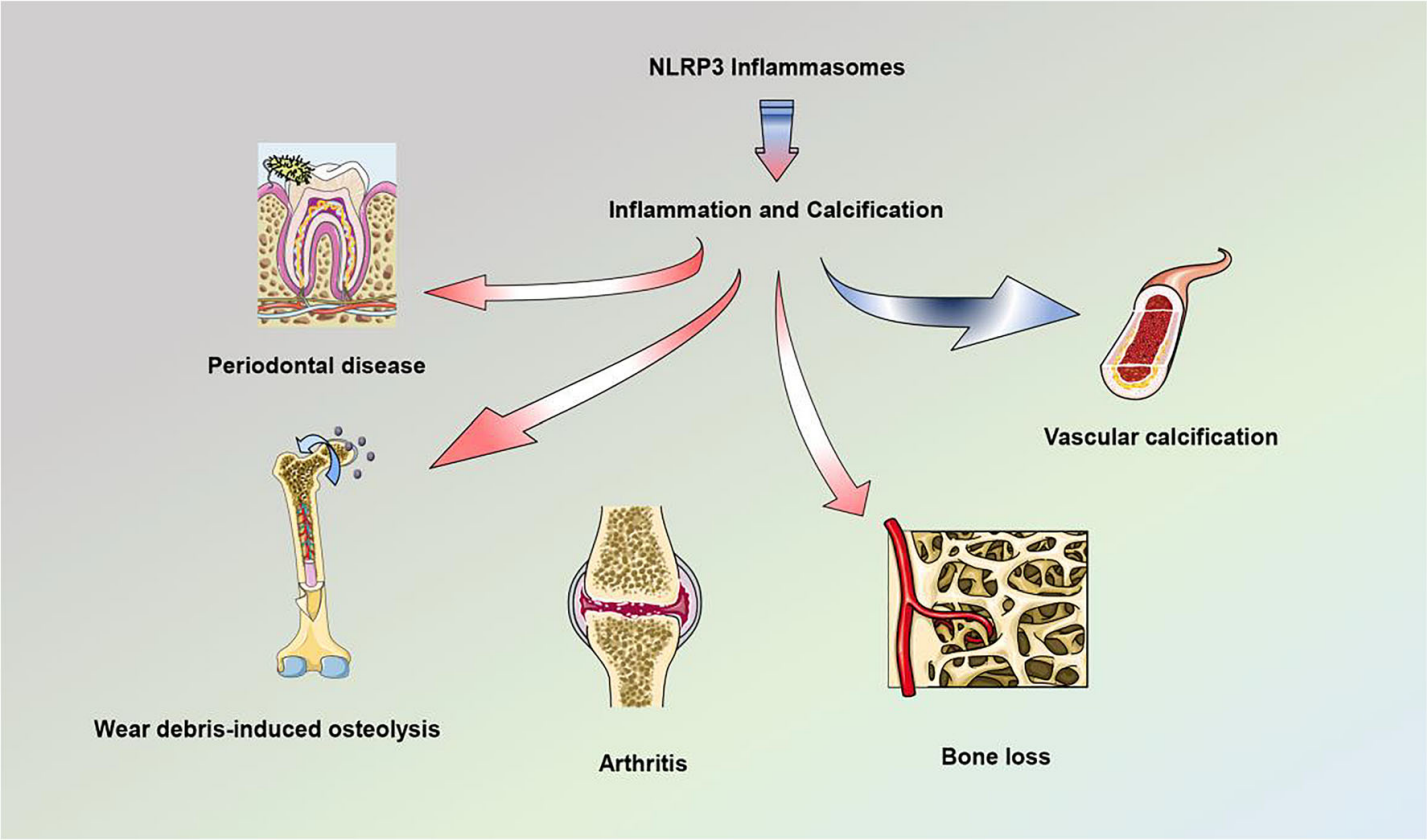
The NLRP3 inflammasome significantly contributes to diseases such as bone loss, arthritis, osteomyelitis, periodontal disease, and vascular calcification by regulating inflammatory response and calcification. Overexpression of NLRP3 inflammasome exacerbates inflammatory osteolysis and inhibits calcium deposition in metabolic bone disease. Bone formation *in vivo* regularly requires neovascularization to facilitate blood perfusion, but NLRP3 inflammasome suppresses the expression of angiogenesis-related genes. Pro-inflammatory cytokines promote calcification of vascular endothelial cells in blood vessels. These findings present potential and relevant treatment strategies for targeted bone diseases and vascular calcification.

Vascular calcification, the deposition of hydroxyapatite minerals in the arterial wall, is a common pathological manifestation of atherosclerosis, diabetic vascular disease, vascular injury, chronic kidney disease, and aging [25–27]. Vascular calcification is also essentially affected by the mechanism of osteogenesis and osteoclast balance, which is similar to the process of bone development. Vascular smooth muscle cells (VSMCs) are the predominant cell types that constitute the arterial wall and are instrumental in the regulation of vascular tone and maintenance of the dynamic stability of blood pressure [28]. In the basal state, VSMCs exhibit a contractile phenotype. In the vascular calcification model, VSMCs are transformed from a contractile phenotype to a synthetic phenotype and eventually develop into an osteoblast-like cell phenotype [29]. Previous experiments have confirmed that VSMCs express bone formation–related transcription factors such as Runx2, Osterix, Msx2 under high phosphorus, high glucose (HG), inflammatory factors, oxidative stress, and other calcification stimulating factors [30]. The OPG/RANKL/RANK signaling pathway is one of the significant pathways that regulate bone metabolism. A combination of RANK and RANKL can promote vascular calcification, whereas OPG inhibits vascular calcification [31]. Previous studies have revealed that the activation of NLRP3 inflammasome can accelerate vascular calcification *in vivo* and *in vitro*. The present study reviews the known and potential roles of NLRP3 inflammasome in bone disease and vascular calcification caused by sterile or non-sterile inflammation (Fig. 2).

## MECHANISMS OF BONE DISEASES AND VASCULAR CALCIFICATION THAT ARE RELEVANT TO THE NLRP3 INFLAMMASOMES

Evidence suggests that the NLRP3 inflammasome is implicated in a wide range of diseases of bone diseases and vascular calcification (Table 1).

**Table 1:** The Regulation of NLRP3 Inflammasome in Bone Diseases and Vascular Calcification

Disorders	Intervention	Mechanism	Target of action	Effect	Ref
Osteoporosis and inflammatory osteolysis	CGRP	ROS/NF-κB/NLRP3	IL-1β	OB	[32]
	IFN-λ1	JAK/STAT/NLRP3	IL-1β, IL-6, TNF-α	OC	[33]
	Melatonin	Wnt/β-catenin/NLRP3	IL-1β	OB	[34]
	—	RIPK1/RIPK3/MAPK/NF-κB/NLRP3	IL-1β, IL-18	OC	[35]
	Irisin	Nrf2/NLRP3	Bcl-2	Osteoblast apoptosis and OP	[36]
	shRNA-NLRP3	—	IL-1β	OB	[37]
	Propofol	MAPK/NFκB/NLRP3	IL-1β, IL-6, TNF-α, COX2	Anti-inflammatory	[38]
	ARTD1 ^D214N^mice	NLRP3	—	OC	[39]
	IL-18BP	NLRP3	IL-2, IL-6, IL-17, IFN-γ, TNFα	OP	[40]
	Glyburide	NLRP3	IL-1β, IFN-γ, TNF-α, IL-6	OC	[41, 42]
Arthritis	IL10-KO	NLRP3	IL-1β, IL-33	Loss of cartilage and OC	[43]
	Gentiopicroside	ROS/NF-κB/NLRP3	—		[44]
	Nucleosides	TREM/NLRP3	IL-1β		[45]
Osteomyelitis	Pstpip2^cmo^mice	SYK/NF-κB	IL-1β	Anti-inflammatory	[46]
	dsRNA	NLRP3	IL-1β, IL-18	Osteoblast cell death	[47]
Periodontal disease	Dioscin	ROS/NF-κB/NLRP3	IL-1β	OB and anti-inflammatory	[48]
	Glibenclamide/Glimepiride	NLRP3	IL-1β	Anti-inflammatory	[49]
	Metformin	NEK7/NLRP3	GSDMD	Pyroptosis	[50]
	PKR	NFκB/NLRP3	—	*Porphyromonas gingivalis*–infected osteoblasts	[51]
Wear debris–induced osteolysis	NMN	TXNIP/NLRP3	IL-1β, IL-18	Anti-inflammatory	[52]
Vascular calcification	6-Shogaol	Akt/NLRP3	IL-1β	Vascular calcification related diabetes Calcification of	[53]
	CAPE	Akt/ERK/ NF-κB/NLRP3	—	AVICs	[54]
	Trimethylamine N-oxide	NF-κB/NLRP3	IL-1β	Vascular calcification related CKD	[55]
	Puerarin	ROS/NF-κB/NLRP3	IL-1β, IL-6, IL-18, TNFα	Calcification of VSMCs	[56]
	β-hydroxybutyrate	NLRP3	IL-1β	Nephrocalcinosis-related CKD	[57]

*OB* osteoblastogenesis, *OC* osteoclastogenesis, *OP* osteoporosis, *CKD* chronic kidney disease

### Osteoporosis and Inflammatory Osteolysis

Inflammasomes are associated with osteoporosis caused by aging, diabetes, and menopause. Chronic inflammation is manifested through continuous secretion of inflammatory mediators and other factors, which consequently cause the destruction of bone tissues and impaired bone homeostasis. Several studies have established that inhibiting inflammation within the fracture microenvironment can promote osteogenic differentiation of stem cells. The level of pro-inflammatory cytokines is increased in the absence of estrogen. Blocking TNF-α or IL-1β in postmenopausal women causes a reduction in the expression of bone resorption markers [58]. Therefore, the inhibition of TNF-α or NLRP3 can prevent bone loss caused by ovariectomy in mice [59, 60]. High glucose in the bloodstream increases bone resorption capacity and efferocytosis of osteoclasts by inducing NOX2-dependent reactive oxygen species (ROS) production and the expression of p-p38-, p-pERK NF-κB-, and NLRP-related proteins in HG-induced osteoclasts, resulting in diabetes-induced osteoporosis in the male rats induced by streptozotocin (STZ) [61]. Aging may be associated with a low level of chronic inflammation indicated by elevated levels of IL-1β, IL-6, and IL-18. NLRP3 inflammasome regulates age-related inflammation and bone loss in peripheral tissues of mice, although the underlying mechanism is still unclear [62–64]. LPS is the most common form of stimulus that induces inflammation. Previous studies have revealed that the expression of NLRP3 and caspase-1 increases after administration of LPS in human umbilical cord mesenchymal stem cells (HUCMSCs) and can inhibit osteogenic differentiation and enhance adipogenic differentiation [65]. The inhibition of NLRP3 inflammasome with MCC950 can ameliorate migration of osteoblasts and restore the expression of osteogenic-related proteins [66]. The inhibition of NLRP3 inflammasome by shRNA targeting NLRP3 can also increase the expression of osteogenic markers and enhance healing of alveolar bone defects in diabetic rats [37].

Therefore, it is imperative to identify suitable NLRP3 inflammasome inhibitors or related signaling pathways that inhibit osteoclast activity and bone loss. The calcitonin gene–related peptide (CGRP) inhibits the expression of NLRP3 and IL-1β in mouse osteoblasts in a dose-dependent manner to promote osteogenic differentiation, and reduce the production of ROS [32]. Dendritic cell–derived IFN-λ1 inhibits both LPS and RANKL-induced osteoclastogenesis and release of pro-inflammatory cytokines in RAW264.7 cells by preventing the classic NF-κB signaling pathway that is associated with NLRP3 inflammasome and activating the JAK/STAT signaling pathway [33]. Similarly, irisin inhibits NLRP3 and IL-1β by upregulating Nrf2, thereby inhibiting apoptosis of osteoblasts in ovariectomized rats [36]. Bone matrix components such as bone particles can considerably promote osteoclast production in wild mice, but this effect is effectively eliminated in Nlrp3^−/−^ mice [60]. Moreover, poly ADP-ribosylation, also known as PARylation is another biochemical modification that has a potential impact on osteoclasts. Poly ADP-ribosylation is catalyzed by ADP-ribosyltransferase diphtheria toxin (ARTD) or poly ADP-ribose polymerase (PARP). PARP is the cleaving substrate of caspase-1. ARTD1, also known as PARP1, is associated with DNA repair and cell proliferation. Studies have demonstrated that activation of the NLRP3 inflammasome can trigger a cascade reaction, causing fragmentation of ARTD1. In the case of Artd1^D214N/D214N^ mice with uncleaved ARTD1, it was established that high bone mass in mice was not necessarily associated with increased osteogenic activity, but it was associated with damage to the intrinsic ability of osteoclastogenesis [39, 67]. The Wnt/β-catenin pathway is a classic signaling pathway that promotes osteogenic differentiation. Melatonin inhibits the activation of NLRP3 inflammasome by mediating the Wnt/β-catenin pathway in BMSCs from ovariectomized (OVX) mice, thereby enhancing osteoporosis in OVX-treated mice [34]. This finding is consistent with previous research. However, Huang et al. found that β-catenin promotes NLRP3 inflammasome activation in mouse peritoneal macrophages independent of its transcriptional activity and has no effect on the expression of NLRP3 components, while promoting the NLRP3 inflammasome assembly [68]. Results revealed that the Wnt/β-catenin signaling pathway had a distinct effect on NLRP3 inflammasome. We hypothesized that β-catenin affects NLRP3 inflammasome in different ways. The Wnt signaling pathway targets activation and assembly of the NLRP3 inflammasome or its transcriptional activity. Additionally, the application of different types of inhibitors to various types of cells may cause the difference. For example, β-catenin inhibitor DKK1 is applied to mouse mesenchymal stem cells [34], but inhibitors XAV939 and siRNA-β-catenin are applied to primary peritoneal macrophages of mice [68]. Glyburide, which has been demonstrated to be an effective NLRP3 inflammasome inhibitor, can significantly reduce the expression of IFN-γ, TNF-α, and IL-6 in the STZ-induced male mice [42] and ameliorate bone destruction in male rats caused by traumatic occlusion [41]. IL-18BP is a natural antagonist of IL-18, and IL-18 is another significant effector of NLRP3 inflammasome. The modulation of the NLRP3 inflammasome pathway by IL-18BP enhances osteoblast differentiation and restores trabecular bone microstructure in OVX mice [40]. Researchers generated a mouse line in which the NLRP3 locus was humanized by syntenic replacement to further explore the role of NLRP3 inflammasome *in vivo*. Results revealed that both mouse and human NLRP3/Nlrp3 mRNA expression levels exhibited a rapid but transient increase after LPS stimulation. IL-1β was not detected in lysates prepared from the paws of normal mice, but it was increased in samples of mutant animals. Osteoporosis and arthritis were detected in Nlrp3(D305N) mice in comparison with wild type (WT) mice, providing an NLRP3-dependent arthritis model platform for testing therapeutic agents against inflammasomes [69]. Nevertheless, systemic inflammation may indirectly affect bone homeostasis. The present study revealed that systemic inflammation did not occur in mouse osteoclasts specifically expressing NLRP3. The number of osteoclasts did not change, but the bone mass decreased by approximately 50% [70]. Furthermore, NLRP3 inflammasome has different roles in elderly and young individuals. Bone loss in older mice lacking Nlrp3 is enhanced through bone resorption rather than bone formation. Similarly, MCC950 inhibited osteoclast differentiation and reduced caspase-1 activation. This phenomenon was not observed in young mice [71].

Cell death can be classified as apoptosis, necroptosis, pyroptosis, autophagic cell death, and other forms of cell death. Pyroptosis is a newly discovered form of programmed cell death in inflammatory cells. Pyroptosis is chiefly mediated through activation of various caspases including caspase-1 by the NLRP3 inflammasome. Caspase-1 causes shearing of various members of the Gasdermin family including GSDMD, subsequently resulting in cell perforation and death [72]. Pyroptosis occurs rapidly and is accompanied by the release of numerous pro-inflammatory factors in comparison with apoptosis. Treatment with LPS causes the NLRP3 inflammasome to mediate cell death in MG-63 cells and reduce cell migration, resulting in osteogenic dysfunction. Moreover, LPS increases the release of lactate dehydrogenase (LDH) in a time and dose-dependent manner. The inhibitory effect of *N*-acetyl-l-cysteine on ROS attenuated LPS-treated pyroptosis and enhanced cell migration in osteoblasts [66]. RIPK1 and RIPK3 are two homologous serine/threonine kinases, which are key elements in the mediation of cell death [73]. A series of innate immune receptors such as TNFR, IFNR, and TLR activate RIPK1 and RIPK3 and then initiate NF-κB/MAPKs signaling pathway and NLRP3 inflammasome in RANKL-treated bone marrow macrophages (BMMs) [35].

Previous researchers have attempted to conduct studies on the NLRP3 inflammasome in patients with fractures. The studies indicated that the levels of TNF-α, IL-1β, and IL-6 in the serum of children with ankle fractures were significantly higher than in healthy children. Furthermore, with reference to inflammation induced by bradykinin (BK) in MG-63 cells, treatment with propofol can decrease BK-induced p-p38, p-p65, COX-2, and NLRP3 in a dose-dependent manner [38]. This indicates that propofol has an effect on the MAPK/NF-κB/NLRP3 signaling pathway. The researchers did not further investigate the relationship of propofol and elevated inflammatory mediators in children with fractures possibly because of safety concerns. Similarly, the expressions of NLRP3 and IL-1β in the cartilaginous endplate tissues of patients with low back pain and Modic changes on MRI were upregulated in comparison with vertebral burst fractures that were not observed in MRI [74]. Notably, the NLRP3 inflammasome was detected in degenerated human tissues.

### Arthritis

Inflammasomes are activated in several types of arthritis. IL-10 is an anti-inflammatory cytokine with crucial immunoregulatory functions, and it inhibits the expression of inflammatory cytokines such as TNF-α, IL-6, and IL-1β in activated macrophages. IL-10 knockout (KO) mice exhibited severe arthritis in a previous study. The expression of IL-1β, IL-33, and NLRP3 in the synovium and the activity of osteoclasts was significantly increased [43]. The expression of NLRP3 inflammasome was upregulated in patients with rheumatoid arthritis (RA) and ankylosing spondylitis (AS), and this was associated with high levels of pro-inflammatory cytokines [75–77]. Suppression of the ROS/NF-κB/NLRP3 axis can effectively regulate inflammatory processes of human RA fibroblast–like synoviocytes (RA-FLS). Correspondingly, gentiopicroside was able to alleviate RA symptoms in rats [44]. The interaction between these inflammatory mediators and other cytokines may cause systemic and focal osteolysis in arthritis. Triggering receptor expressed on myeloid cells (TREM) is a type of immunoglobulin-like receptor on cell surfaces, which performs a negative regulatory role in autoimmunity and inflammation [78]. In mice with type II collagen-induced arthritis (CIA), nucleosides and nucleoside analogs can activate the innate immune system through surface TREM receptors and intracellular NLRP3 inflammasome. This consequently produces IL-1β that stimulates osteoclasts and recruits more preosteoclasts to the joint space to enhance inflammatory osteolysis [45].

### Osteomyelitis

Osteomyelitis regularly causes severe bone destruction. Osteoblasts exposed to the primary pathogen of osteomyelitis are prone to apoptosis. *Salmonella* can induce osteoblast apoptosis by activating NLRP3 and caspase-1 [79]. The products of *Staphylococcus aureus* including α-toxin and Panton-Valentine leukocidin (PVL) stimulate the NLRP3 inflammasome in human phagocytes through K^+^ efflux and cathepsin B activation [80]. Pyroptosis is usually accompanied by the release of numerous pro-inflammatory cytokines which promote the formation of osteoclasts. Therefore, inhibitory effect of cell pyroptosis can restore bone formation characteristics and effectively reduce bone destruction in osteomyelitis. The expressions of pyroptosis-related proteins, NLRP3, caspase-1, and GSDMD were significantly increased in mouse osteomyelitis model induced by *Staphylococcus aureus* and infectious bone fragments of patients with osteomyelitis. NLRP3 and caspase-1 inhibitors both attenuated *S. aureus*–induced pyroptosis in MC3T3-E1 cells and mice [81]. DICER1 is an endoribonuclease that can recognize and cleave dsRNA in cells. *Staphylococcus* infection, inhibition of DICER1, or administration of dsRNA can activate the NLRP3 inflammasome in osteoblasts, increase the expression of IL-18 and IL-1β, and reduce the viability of MG-63 cells. Moreover, the inhibition of caspase-1 can completely block death of human fetal osteoblast cells (hFOB) induced by DICER1 deficiency or dsRNA accumulation [47].

Self-inflammatory reactions of unknown etiology can cause localized chronic nonbacterial osteomyelitis (CNO) or systemic chronic recurrent multifocal osteomyelitis (CRMO). Previous researches on humans have indicated the expression of NLRP3 inflammasome in osteoclasts from bone specimens of patients with CRMO [82]. In animals, the progression of disease in Pstpip2^cmo^ mice is mediated by IL-1β, which is similar to human CRMO [83]. Spleen tyrosine kinase (SYK) performs a key role upstream of caspase-1 and caspase-8, primarily upregulating NF-κB and IL-1β signaling in Pstpip2^cmo^ mice, and induction of chronic multifocal osteomyelitis (CMO). RIPK3 plays a vital role in disease progression. Remarkably, SYK does not activate the NLRP3 inflammasome [46].

### Wear Debris–Induced Osteolysis

Clinically, aseptic loosening is the principal reason that limits the long-term service life of artificial joint prostheses, in which wear particles such as cobalt-titanium-chromium (Co–Ti–Cr) implants activate macrophages to produce pro-inflammatory cytokines around the prosthesis [84]. Osteolysis is considered the most significant factor in the occurrence of aseptic loosening. Abrasion particles can activate the PRR of macrophages to induce activation signal of the NLRP3 inflammasome, thereby producing TNF-α and IL-1β and resulting in osteolysis around the prosthesis. Macrophages induced by Ti particles activate NLRP3 inflammasome to secrete IL-1β and simultaneously trigger the recruitment of neutrophils. The administration of IL-1 receptor antagonist (IL-1RA) alleviates this effect [85]. Blocking DAMP can remarkably reduce the responses of macrophages associated with IL-1β and TNF-α to wear particles compared to blocking TLR4 [86]. In addition, a previous study indicated that Ti particles coated with LTA stimulated macrophages to increase the expression of NLRP3 inflammasome associated with TNF-α and TLR2. Although TNF-α was released, no increased secretion of IL-1β was detected [87]. Macrophages pretreated with LPS can produce IL-1β when engulfing polymethyl methacrylate (PMMA) and Ti particles. Inhibiting the activation of NF-κB induced by PMMA particles can effectively prevent osteoclastogenesis and reduce PMMA-induced inflammation and osteolysis in mice [88]. Inflammatory factors induced by metal components are also regulated by the inflammatory response pathway. Preosteoblasts were placed on a nanoscaled hydroxyapatite–blasted Ti texturing surface, and it was established that sonic hedgehog (SHH) signaling pathway and osteoblast differentiation were positively regulated [89]. However, the interaction between SHH pathway and NLRP3 inflammasome had not been explored. The nicotinamide mononucleotide (NMN) inhibits the expression of inflammatory factors through the TXNIP-NLRP3 inflammasome pathway, significantly reducing aluminum-induced bone damage. The knocking down of TXNIP has a similar protective effect to that of NMN in MC3T3-E1 cells [52].

### Genetic Diseases

Autoinflammatory diseases (AIDs) are a group of hereditary, recurrent, and non-invasive inflammatory diseases, which are largely caused by abnormal expression or clearance of inflammatory factors caused by gene mutations [90]. The clinical manifestations of AIDs such as fever, rash, arthralgia, and arthritis can affect multiple organ systems, and IL-1β is implicated in the occurrence and development of the disease [91]. AIDs include cryopyrin-associated periodic syndrome (CAPS), familial Mediterranean fever (FMF), and NLRP12-related periodic fever among others. CAPS is a genetic disease caused by an autosomal dominant mutation in the NLRP3-NACHT domain. CAPS include neonatal-onset multisystem inflammatory disease (NOMID), familial cold autoinflammatory syndrome (FCAS), and Muckle-Wells syndrome (MWS). Skeletal abnormalities, knee protrusion, and low bone mass are unique features of patients with NOMID [92]. The pluripotent stem cells induced in patients with NOMID have a higher proliferative capacity and higher differentiation potential than normal cells, which is consistent with the tumor-like characteristics of bone growth [93]. Researchers have generated NOMID mice that can integrally express the NLRP3 D301N mutation. NOMID mice exhibited increased neutrophils and serum inflammatory mediators in the blood and knee joints, accelerated bone resorption accompanied by increased osteoclasts, growth retardation, and severe postpartum bone loss [92]. Epiphyseal abnormalities undoubtedly increase the risk of joint instability and osteoarthritis in patients with NOMID [94]. Conversely, the lack of the IL-1 receptor in NOMID mice completely prevents systemic inflammation and various organ diseases including skeletal abnormalities [95]. Previous studies have reported that residual inflammation persists in FCAS mice and MWS mice lacking IL-1 signaling [96, 97]. Nonetheless, other results indicate that the activation of IL-1β by NLRP3 is not the prime driver of skeletal pathology in CAPS. The release of TNF mediated by a mutant gain-of-function NLRP3 inflammasome in the serum of patients with CAPS is not blocked by IL-1RA. By contrast, the neutralization of TNF-α activity improves the inflammatory endpoints of CAPS mice [96]. Excessive activation of pyrin inflammasome caused by MEFV-activating mutations can cause FMF [98, 99]. However, the symptoms of systemic inflammation disappear in cells from *Il1r1*^−/−^, *Asc*^−/−^[100], and *GSDMD* ^−/−^ mice [101]. Approximately 20% of patients suffering from FMF are below 2 years of age, and two-thirds of patients develop FMF disease before the age of 10 years. This feature corresponds to macrophage activation syndrome (MAS). MAS is a common complication of systemic juvenile idiopathic arthritis (sJIA), with the ability to cause osteoporosis and periostitis around the joints. Joint bone destruction can be observed during later stages [102]. Although the level of IL-1β in MAS is usually lower than that of CAPS, the IL-1RA anakinra is effective in the treatment of sJIA. No changes have been detected in the expression of NLRP3-associated transcription products [103, 104].

Gaucher disease (GD) is caused by the accumulation of glucocerebroside in the lysosomes of macrophages in multiple tissues and organs, resulting in the formation of lesions in affected tissues and organs. GD is an autosomal recessive genetic disorder. Most patients have a bone intrusion. A patient with mild GD presents osteopenia, whereas a patient with severe GD presents local osteolysis, pathological fractures, and joint damage [105]. A few children may experience growth retardation. Osteoblasts from patients with GD reveal suppressed osteogenic differentiation. *In vitro* experiments have demonstrated that the conditioned media from culture supernatants of MSCs in patients with GD increases the number of osteoclasts. Moreover, expressions of NLRP3 and PPAR-γ genes were promoted in MSCs of patients with GD [106], indicating that the NLRP3 inflammasome is involved in the negative regulation of bone changes in GD.

### Periodontitis

*Enterococcus faecalis* is the most common pathogen of refractory periapical periodontitis that develops after a root canal treatment. Dioscin, the NLRP3 inflammasome inhibitor, promotes osteogenesis by inhibiting the nuclear transport of NF-κB and the expression of ROS induced by LTA from the *Enterococcus faecalis* [48]. Although glyburide can effectively repair bone defects, its role in inflammation caused by bacteria associated with periodontal disease is slightly different. THP-1 (a human monocyte cell line) differentiates into macrophage-like cells after infection with periodontal pathogens such as *Porphyromonas gingivalis* (*P.g*) and *Fusobacterium nucleatum*. THP-1 induces the secretion of IL-1β, which is inhibited by NLRP3 inhibitor, MCC950 and caspase-1 inhibitor z-YVAD-FMK. Treatment with glibenclamide inhibits the expression of IL-1β in the cell supernatant, but its protein expression is increased in cell lysates, suggesting that glibenclamide can inhibit secretion of IL-1β in THP-1. Glimepiride presents a similar effect, whereas biguanide hypoglycemic drugs such as metformin cannot inhibit secretion of IL-1β [49]. Nevertheless, metformin treatment can ameliorate diabetes mice with experimental periodontitis and inhibit cell pyroptosis through the downregulation of the NEK7/NLRP3/GSDMD pathway [50]. A previous study revealed that PKR was activated in MC3T3-E1 cells infected with *P.g*, which subsequently activated NF-κB to enhance the expression of NLRP3, but shRNA-PKR reversed this effect [51]. Heat-killed *Aggregatibacter actinomycetemcomitans* were injected into the gum tissues of Nlrp3-KO and Casp1-KO mice to induce alveolar bone resorption. Strikingly, bone resorption in Casp-1 KO mice decreased significantly, but the number of osteoclasts increased significantly. Bone resorption in Nlrp3-KO mice did not significantly change in comparison with WT mice [107], implying that caspase-1 is instrumental in modulating inflammation caused by bacteria of periodontal disease, and may be regulated by genes other than NLRP3.

### Vascular Calcification

NLRP3 inflammasome performs a pivotal role in the calcification of vascular smooth muscle cells (VSMCs). When calcification occurs in rat and human VSMCs, the NLRP3 inflammasome is activated. Furthermore, previous research has revealed upregulation of the NLRP3 inflammasome in the popliteal artery specimens from 5 clinical samples [108]. Atherosclerosis of the large and middle arteries or arterial media calcification is a prevalent complication of diabetes. Endothelial cells also undergo osteogenic changes in high-glucose environments. Plasma trimethylamine *N*-oxide (TMAO) and puerarin inhibit calcification of rat VSMCs by targeting the classic NF-κB/NLRP3 inflammatory pathway [55, 56]. The calcification of human aortic smooth muscle cells (HASMCs) is attenuated by 6-Shogaol through inhibition of Akt/NLRP3/IL-1β signaling [53]. Similarly, caffeic acid phenethyl ester (CAPE) inhibits calcification of aortic valve interstitial cells (AVICs) *via* PI3K-AKT/ERK/NF-κB/NLRP3 signaling pathway [54]. Double-stranded RNA (dsRNA) acts as a PAMP in the cytoplasm, thereby triggering an immune response [109]. Polyinosinic-polycytidylic acid [poly(I:C)] is a synthetic mimic of dsRNA that can be recognized by TLR3 and inflammatory stimulating factors. Poly(I:C) upregulates the production of BMP-2, ALP and promotes the formation of calcium deposits in AVICs through mediation of the TLR3/NF-κB/ERK pathways. However, dsRNA has no significant effect on the NLRP3 inflammasome in AVICs [110].

**Fig. 3:**
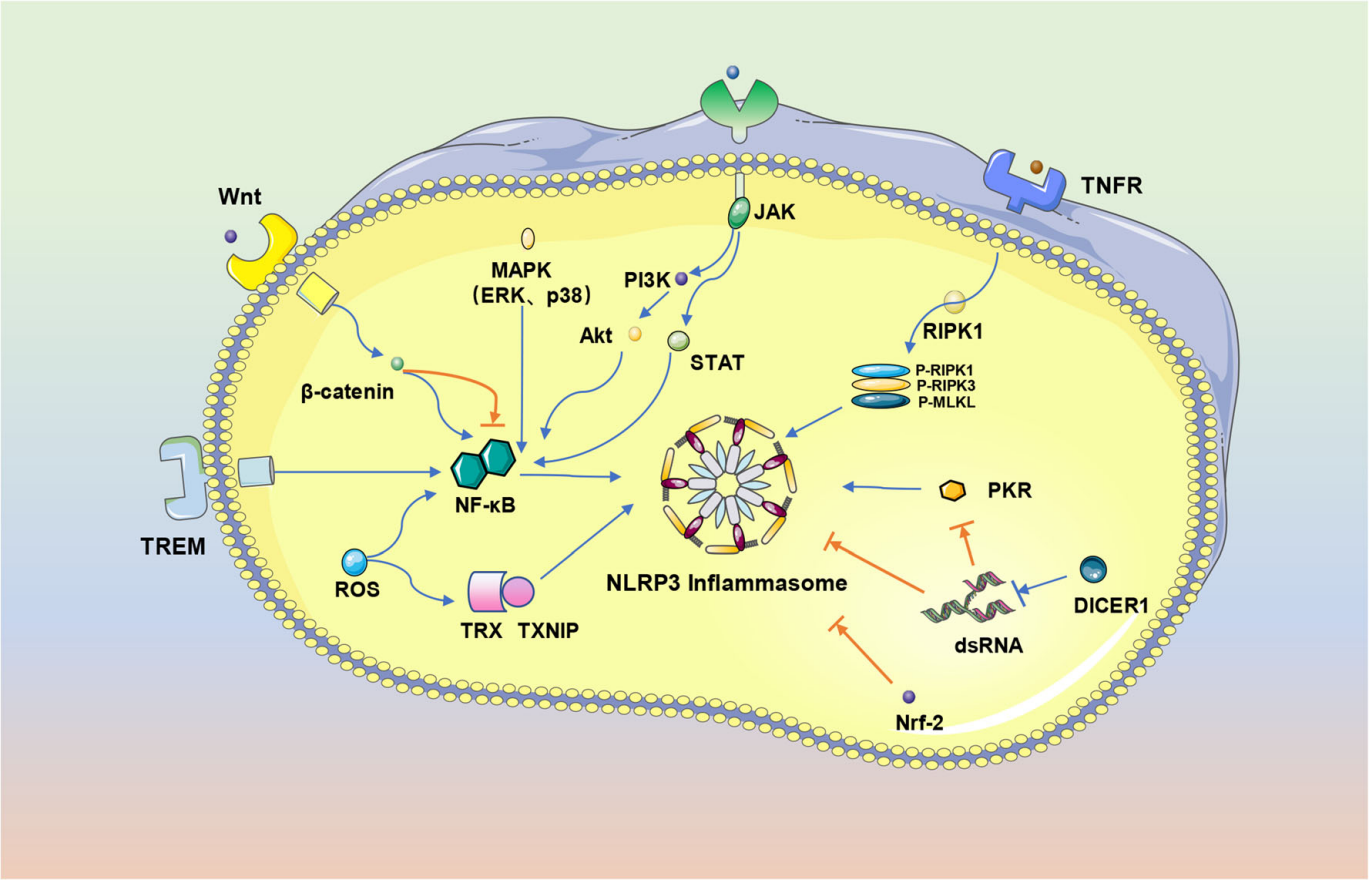
Effects of signaling pathways and gene regulation on NLRP3 inflammasome. The regulatory effects of different signaling pathways on NLRP3 inflammasome in diseases associated with bone loss and vascular calcification were evaluated. JAK positively regulates NLRP3 inflammasome through the PI3K/Akt and JAK/STAT pathways. The Wnt pathway exhibits a different regulatory pathway for NLRP3 inflammasome, which may differ from the effect of β-catenin on NLRP3 inflammasome. MAPK is a common upstream pathway node of NF-kB and several studies have established that MAPK has a similar regulatory effect to the classic NF-kB/NLRP3 inflammatory response pathway. Numerous studies have established that Nrf2 can indirectly inhibit the MAPK signaling pathway, thus inhibiting the production of inflammatory mediators. RIPK1 undergoes polyubiquitination under TNF stimulation. Subsequently, RIPK1 and RIPK3 are phosphorylated to form a necrosome complex and promote necroptosis by stimulating NLRP3 inflammasome. Thioredoxin-interacting protein (TXNIP), a multifunctional protein involved in the maintenance of homeostasis and is isolated from thioredoxin (TRX) in response to ROS, activates the NLRP3 inflammasome. TREM is a glycoprotein molecule on the plasma membrane, which works together with the TLR of monocyte-macrophages to activate NLRP3 inflammasome and enhance pro-inflammatory effect. Previous studies have established that PKR is activated by binding to dsRNA derived from viruses. DICER, a member of the RNase III family, specifically recognizes and cleaves dsRNA. Activated PKR induces immune response through phosphorylation of eIF2α.

For a long time, vascular calcification is a recognized risk factor for cardiovascular disease morbidity and mortality. As mentioned above, ectopic calcification is a pathological phenotype with high incidence, which is related to chronic kidney disease, atherosclerotic cardiovascular disease, and diabetes. However, the role of NLRP3 inflammasome in human kidney disease is still indeterminate. Research conducted in the past has revealed that NLRP3 of lupus nephritis and non-diabetic chronic kidney disease (CKD)in human are substantially upregulated [111]. The NLRP3 inflammasome inhibitor, β-hydroxybutyrate, alleviates CKD associated with renal calcification in mice [57]. The most extensively studied component of the NLRP3 inflammasome associated with kidney disease is IL-18. In addition to mediating acute forms of kidney injury and diseases, the IL-1/IL-18 axis can cause CKD-related complications such as acute atherosclerosis and vascular calcification [112]. Inflammation contributes to vascular calcification, and IL-18 causes inflammation-related vascular damage, atherosclerotic plaque formation, and plaque instability by producing INF-γ [113–115]. Although IL-1β is considered to be a key inflammatory factor activated by the NLRP3 inflammasome, the IL-1 inhibitor anakinra has no effect on related indicators of renal calcification in mice. This indicates that NLRP3 inflammasome and ASC promote the development of CKD associated with renal calcification but have no effect on the secretion of IL-1β induced by the inflammasome (Fig. 3) [57].

## THERAPEUTIC PERSPECTIVE

Bone loss and vascular calcification stem from systemic or local inflammation. Anti-osteoporosis drugs can effectively prevent metabolic bone diseases, but they do not affect inflammatory process. Bone healing process is affected by the inflammatory environment surrounding the bone. Osteogenic differentiation and neovascularization promote fracture healing. Evidence shows that the inhibition of TLR4/NF-κB/NLRP3 inflammation signaling pathway enhances angiogenesis; poor blood perfusion caused by vascular calcification can adversely affect bone formation [116]. Moreover, the VSMC phenotypic change related to calcification may also have significant differences in the factors that drive calcification at different anatomical sites. For instance, patients with renal failure manifest accelerated medial calcification, but the calcification of both the media and intima may be accelerated in diabetes [117]. The blockade of branching arteries belonging to the abdominal aorta cause lumbar spine ischemia due to poor perfusion, leading to degeneration and asymptomatic vertebral fractures [118]. A 10-year prospective study found that older women with significant abdominal aortic calcification had a higher risk of fracture [119]. In addition, atherosclerotic lesions release local and systemic osteochondrogenic factors, thereby affecting local and systemic bone homeostasis [120, 121]. Recent epidemiological studies have shown that there is a close relationship between bone loss and vascular calcification, and vascular calcification is not related to age [122]. Although therapies that blocking IL-1 can protect bones from destruction by inhibiting inflammation as evidenced in various animal models, the efficacy of such therapies in human has been inconsistent. Therefore, it is crucial to design suitable inhibitors or gene regulatory network to inhibit inflammasome. Such strategies can inhibit inflammation and osteoclast maturation and prevent vascular calcification. Several issues remain unresolved concerning the role of the inflammasome in bone and blood vessels. For instance, (1) Are pathways that inhibit inflammasome restricted to assembly, activation, or other reaction mechanisms? (2) Is the role of the inflammasome in bone functions limited to the maturation of IL-1β, IL-18, and GSDMD? (3) The effective strategies with the purpose of reducing the activity of osteoclasts or inhibiting osteoblast apoptosis by blocking the NLRP3 inflammasome and integrating several signaling ways such as Wnt/β-catenin pathway, MAPK pathway, and SHH pathway are required to explore. (4) Little is known on how to target the NLRP3 inflammasome to simultaneously weaken inflammatory response in the osteolysis and vascular endothelium. (5) Research is advocated to design suitable materials with good drug release dynamics to facilitate local delivery of anti-inflammatory drugs. This will prevent the occurrence of aseptic inflammation on the surface of joint replacements. Findings answers to these questions will lead to the development of drugs targeting the NLRP3 inflammasome as a therapeutic approach to treat bone disease and vascular calcification.
